# Fixed-Life or Rechargeable Battery for Deep Brain Stimulation: Preference and Satisfaction in Chinese Patients With Parkinson's Disease

**DOI:** 10.3389/fneur.2021.668322

**Published:** 2021-06-15

**Authors:** Xian Qiu, Tingting Peng, Zhengyu Lin, Kaiwen Zhu, Yuhan Wang, Bomin Sun, Keyoumars Ashkan, Chencheng Zhang, Dianyou Li

**Affiliations:** ^1^Department of Nursing, Ruijin Hospital, Shanghai Jiao Tong University School of Medicine, Shanghai, China; ^2^Department of Neurosurgery, Ruijin Hospital, Shanghai Jiao Tong University School of Medicine, Shanghai, China; ^3^Department of Neurosurgery, King's College Hospital, London, United Kingdom

**Keywords:** deep brain stimulation, movement disorders, patient satisfaction, Parkinson's disease, rechargeable implantable pulse generator

## Abstract

**Introduction:** DBS is a widely used therapy for PD. There is now a choice between fixed-life implantable pulse generators (IPGs) and rechargeable IPGs, each having advantages and disadvantages. This study aimed to evaluate the preference and satisfaction of Chinese patients with Parkinson's disease (PD) who were treated with deep brain stimulation (DBS).

**Materials and Methods:** Two hundred and twenty PD patients were treated with DBS and completed a self-reported questionnaire to assess their long-term satisfaction and experience with the type of battery they had chosen and the key factors affecting these choices. The survey was performed online and double-checked for completeness and accuracy.

**Results:** The median value of the postoperative duration was 18 months. The most popular way for patients to learn about DBS surgery was through media (79/220, 35.9%) including the Internet and television programs. In total, 87.3% of the DBS used rechargeable IPGs (r-IPG). The choice between rechargeable and non-rechargeable IPGs was significantly associated with affordability ($\chi_{\left(1\right)}^{2}$ = 19.13, *p* < 0.001). Interestingly, the feature of remote programming significantly affected patients' choices between domestic and imported brands ($\chi_{\left(1\right)}^{2}$ = 16.81, *p* < 0.001). 87.7% of the patients were satisfied with the stimulating effects as well as the implanted device itself. 40.6% of the patients with r-IPGs felt confident handling devices within 1 week after discharge. More than half of the patients checked their batteries every week. The mean interval for battery recharge was 4.3 days. 57.8% of the patients spent around 1 h recharging, and 71.4% of them recharged the battery independently.

**Conclusions:** Most patients were satisfied with their choice of IPGs. The patients' economic status and the remote programming function of the device were the two most critical factors in their decision. The skill of recharging the IPG was easy to master for most patients.

## Introduction

Deep brain stimulation (DBS) is a well-established neurosurgical treatment for advanced Parkinson's disease (PD), which achieves its therapeutic effects by the delivery of continuous electrical current to the target structure, circuitry, or network implicated in disease pathophysiology. The electric power comes from an implantable pulse generator (IPG), which was initially designed to be non-rechargeable. Depending on the patient's diagnosis and DBS parameter settings, the fixed-life batteries need to be replaced 3–5 years after implantation. Battery depletion is the most common reason for additional surgery in DBS patients (1, 2). Although IPG replacement is a minor surgical procedure compared to primary brain surgery, multiple IPG replacements over the course of the patient's treatment and illness can pose an increased health risk, such as an elevated post-implantation infection risk and wound healing problems (3).

The rechargeable-IPG was developed in 2008 and outperformed the IPG with fixed-life batteries in various ways (4). First, patients with DBS need fewer IPG replacement surgeries due to battery depletion, thereby reducing surgical-related health risks. Second, rechargeable batteries are less subject to induce unexpected clinical changes, such as a worsening of PD symptoms, whereas the current of fixed-life batteries gets irregular as they are gradually exhausted. Third, rechargeable batteries are smaller in size. The frequency and duration of the IPG battery recharging procedure depend on the power of the DBS needed to manage the PD symptoms of a patient. However, patients are required to check the battery status and need to regularly recharge the battery using a handheld device. The recharging procedure, though not difficult to perform by healthy persons, can be challenging for patients with PD as most of them are elderly with various levels of motor and cognitive deficits (5).

Other technological advances during the past decade have also been incorporated into present-day DBS systems to provide clinicians with more flexible and personalized treatment options. As a result, neurologists and neurosurgeons are facing the challenge of selecting the most appropriate DBS system and treatment for a given patient. They need to make a clinical decision based on many factors, such as lead geometry, the specific DBS target, the amount of current needed for the patient, and the nature of the programming platform of different device brands (6). Other relevant factors include the patients' overall health status, medical history, level of social support available, socioeconomic status, the health care insurance plan, and their psychomotor ability both to deal with the IPG battery recharging procedure and to use modern interactive devices.

The preference of the patients and concerns about how the DBS treatment may impact their daily activities have received little attention in previous studies (5). Most studies have focused on DBS safety and efficacy, but the patient's perspective on the DBS treatment delivered and the reasons for choosing or accepting a specific DBS system remain an important but under-recognized topic. For example, only a few studies performed in Europe have demonstrated that patients with PD preferred rechargeable IPG batteries over fixed-life batteries (7–9). This situation is problematic because patient preference and satisfaction are important for treatment acceptance, treatment compliance, and the further development of next generation of DBS systems. This study aimed to shed more light on patients' perspectives, including their IPG preference and the reasons that drive their choice (e.g., patient affordability, DBS, and IPG device features) of either rechargeable or fixed-life IPG batteries. We also explored preferences for the use of either an imported brand or domestic rechargeable IPG. To address these questions, we examined patient satisfaction and IPG choices in a large cohort of Chinese patients with PD treated with DBS.

## Materials and Methods

### Participants

A total of 768 patients diagnosed with idiopathic PD who underwent DBS surgery at our hospital were contacted and invited to participate in this study. At the time of their DBS surgery, the patients had been offered a choice of receiving an IPG that was either non-rechargeable (nr-IPG) or rechargeable (r-IPG). They were also given a choice of receiving either an imported (Medtronic) or domestic (PINS and SceneRay) brand IPG. Each IPG option had been fully explained and discussed with patients. In China, the costs of surgery were primarily covered by the patient's health insurance, but the costs of DBS hardware were only partly covered. In general, r-IPGs were substantially more expensive than nr-IPGs and international IPG devices were slightly more expensive than domestic made devices. An illustration of the international and domestic brands of IPG devices used in this study can be found in Supplementary Figure 1. The study was approved by the Ethical Committee of Ruijin Hospital. Written informed consent was completed by all participants.

### Survey Questionnaire

An internet-based survey questionnaire (powered by www.wjx.cn) was developed to evaluate the patients' perceptions, expectations, and experiences with the DBS treatment and the IPG device they chose (r-IPGs vs. nr-IPGs and international vs. domestic brands). The questionnaire was designed with reference to the work of Jakobs et al. (5), and were adjusted according to the situation of Chinese patients. The questionnaire focused on the patients' satisfaction with the DBS treatment and the IPG of choice as well as four aspects of possible concern (patient affordability, prospect of further required surgeries to replace the battery, requirement for recharging the battery, and battery size) and the sources of information (e.g., clinical advice, electronic media) the patients considered in making decisions. Several extra questions were included in the questionnaire for patients with r-IPGs to collect their experiences with the battery recharging procedure, including how often they checked the battery capacity and the frequency, duration, and ease of recharging, as well as the occurrences that they forgot or were unable to recharge. The patients indicated their answers using a forced-choice dichotomous format or a 4- or 5-point Likert scale. The questionnaire was distributed via the online chat platform WeChat. Participants completed the questionnaire after having received at least 8 months of DBS treatment. In most cases, it took no more than 30 min to complete the questionnaire.

### Data Processing and Statistical Analysis

The survey question answers were first organized into dichotomous variables (e.g., sex, satisfaction rate), categorical nominal variables (e.g., sources of information used by patients to make their choice), categorical ordinal variables (e.g., patient affordability, frequency, and duration of recharging procedure, frequency of battery capacity checks), and continuous variables (e.g., age). We then computed descriptive statistics consisting of frequencies and percentages, measures of central tendency (median or mean), and measures of variation [interquartile range (IQR) or standard deviation].

Initially, two separate one-sample binomial tests of proportions were performed to ascertain that the patients' choice-preference decisions for r-IPGs or nr-IPGs, as well as for an international or domestic device, which were not made at random but differed significantly from chance (50%).

Fisher's exact test was performed to assess the significance of the association between patient satisfaction rate (satisfied vs. unsatisfied) and the type of IPG (r-IPG vs. nr-IPG). The patient's affordability or budget consisted of 4 ordinal categories (<100,000 RMB, 100,000–200,000 RMB, 200,000–300,000 RMB, and >300,000 RMB). The Fisher-Freeman-Halton Test was used to assess the relationship between patient's affordability and the choice of r-IPG vs. nr-IPG.

Fisher's exact test to assess the relationship between affordability and the choice of r-IPG vs. nr-IPG. We used Pearson's or Yates' continuity corrected Chi-squared test to evaluate the influencing factors for patients' choices of international vs. domestic manufacturers in the r-IPG group, and to compare the satisfaction rate between patients with r-IPG and nr-IPG. A *p*-value < 0.05 was considered significant. Data were analyzed with SPSS (version 23.0; Amonk, NY: IBM). Continuous variables were expressed as Mean ± SD or median value with interquartile range (IQR). Categorical variables were presented as frequencies (%).

## Results

### Patient Sample Characteristics

Two hundred and twenty patients with PD (135 men, 85 women; age: 62.8 ± 9.8 years) out of 768 patients contacted responded to the survey, yielding a response rate of 28.6%. The median duration of postoperative DBS treatment was 18 (IQR: 8–36) months (Table 1).

**Table 1:** Patient sample characteristics (*N* = 220).

**Characteristics**	**Total**	**r-IPG**	**nr-IPG**
**Gender**			
Men (*n*, %)	135 (61%)	114 (59%)	21 (75%)
Women (*n*, %)	85 (39%)	78 (41%)	7 (25%)
**Age, year (mean** **±** **SD)**	62.8 ± 9.8	62.5 ± 9.8	64.4 ± 10.0
**Follow-up, month (median, IQR)**	18 (8–36)	19 (9–38)	15.5 (5.25–26.5)
**Origin of DBS system manufacturer**			
International (Medtronic)	142	136	6
Domestic (PINS or SceneRay)	78	56	22

### Patient Preference

In the patient sample (*n* = 220), 192 (87%) patients used r-IPGs (Table 1). The remaining 28 (13%) patients chose to use nr-IPGs for their DBS treatment. In addition, 142 (65%) patients chose a device from an imported manufacturer (Medtronic), whereas 78 (36%) patients preferred a device from a domestic manufacturer (PINS or SceneRay). Two one-sample binomial tests of proportions (expressed in percentages) confirmed that the patients' choice-preference decisions (i.e., 87% favoring r-IPGs and 65% international devices) were significantly different from that could be expected by chance (50%) (*z* = 8.8 and 11.0, both *p* < 0.001).

### Patient's Sources of DBS Information

The two most common reasons for patients' preference-choice and making a decision prior to surgery were digital media (web sites, television) (36% of all patients) and primary care physicians who referred the patients to our DBS center (36%). A substantial portion of patients (26%) reported having gained information about DBS by word of mouth from other patients with PD treated with DBS in our center.

### Factors Affecting Patient's Choice for r-IPG or nr-IPG

**Figure 1:**
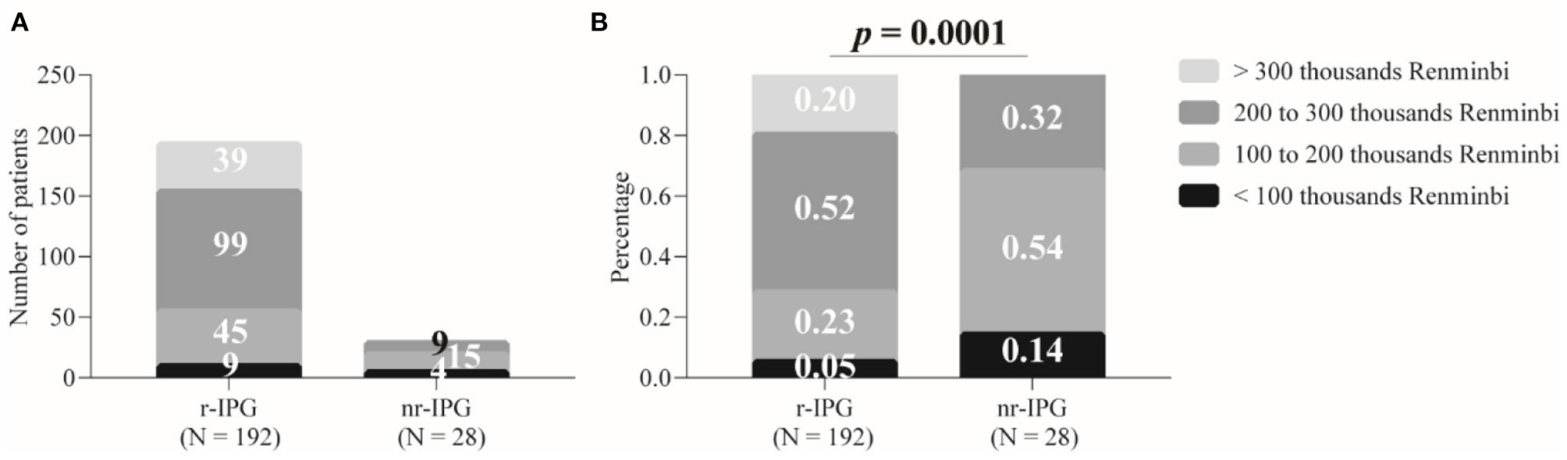
Patient budgets for deep brain stimulation and implanted pulse generators (IPGs). The budgets are divided into four levels. Data are presented as either absolute numbers **(A)** or percentages **(B)**. A *p*-value < 0.05 is considered statistically significant. r-IPG, rechargeable IPG; nr-IPG, non-rechargeable IPG.

We evaluated four factors (patient affordability, prospect of further required surgeries to replace the battery, requirement for recharging the battery, and battery size), which the patients might have considered when choosing a specific IPG. The affordability consisted of four ordinal categories (<100 thousand RMB, 100–200 thousand RMB, 200–300 thousand RMB, and >300 thousand RMB) (Figure 1). A 4 × 2 Chi-squared test to assess the association between patients' affordability and patients' choice could not be performed because one cell was empty in the contingency table analyzed (i.e., there were no patients with the highest budget available who had chosen for an nr-IPS) (Figure 1A).

Nevertheless, a two-sample binomial test of proportions showed that patients with the two highest personal budgets were over-represented among the patients who had chosen an r-IPG (72% of the 192 patients) and under-represented among the patients who opted for an nr-IPG (32% of the 28 patients) (*z* = 4.2, *p* < 0.0001) (Figure 1B). Alternatively, patients with the two lowest budgets were under-represented among the patients who opted for an r-IPG (28%) and over-represented among the patients who chose to use an nr-IPG (68%) (*z* = 4.2, *p* < 0.0001). These results indicate that a significant association existed between patient affordability and choice to use either an r-IPG or an nr-IPG device.

**Figure 2:**
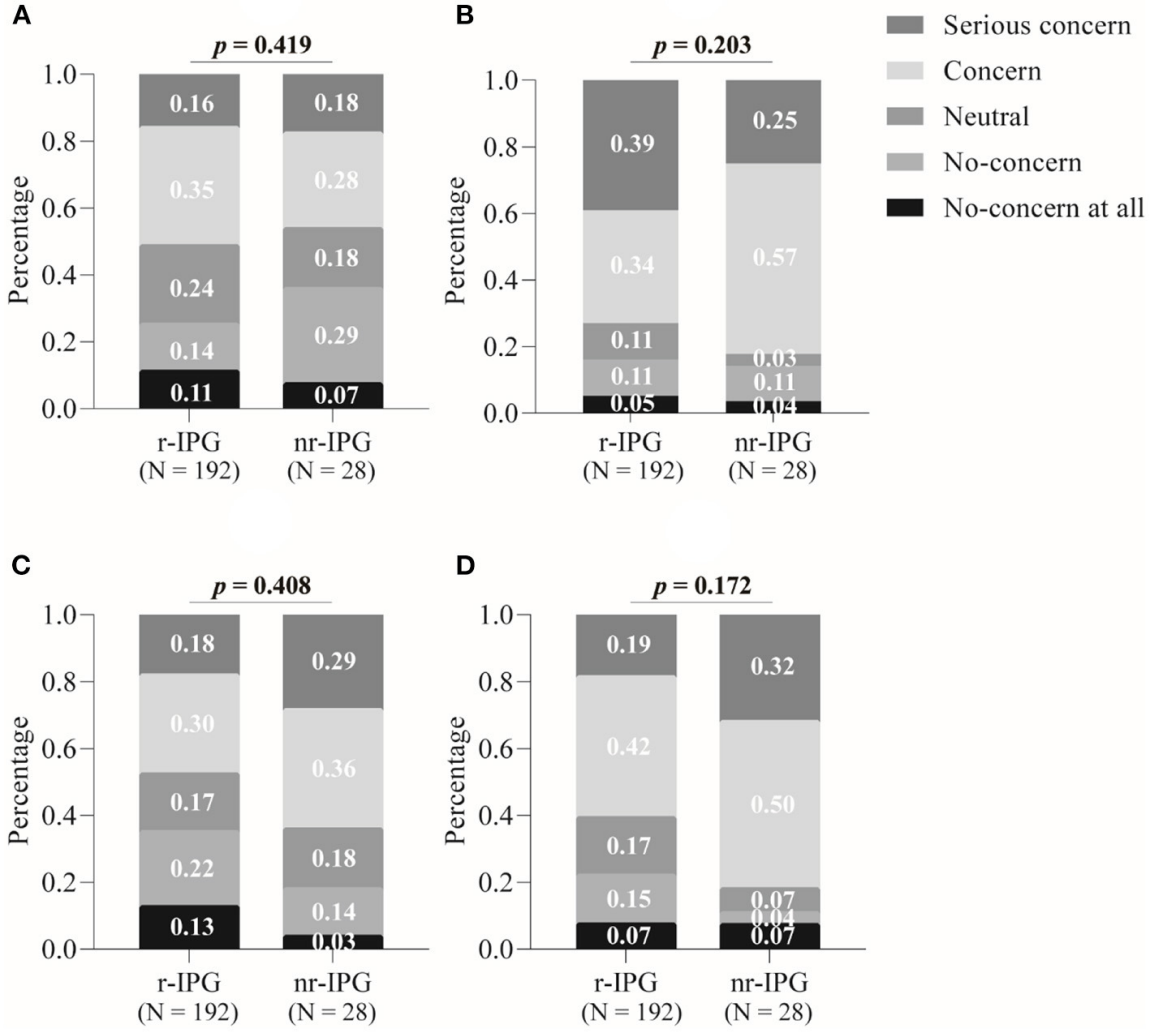
Factors influencing patient's choice between a rechargeable (r-IPG) and non-rechargeable (nr-IPG) implanted pulse generator. **(A)** Battery size; **(B)** the need for further surgery to replace the battery; **(C)** the need for recharging the battery; and **(D)** economic issues. Patients' attitudes toward these factors were divided into five levels in the questionnaire: “No concern at all”, “No concern”, “Neutral”, “Concern”, and “Serious concern”. Data are presented as percentages. A *p*-value < 0.05 is considered statistically significant.

In response to the survey questions about the factors that were of concern for patients' in making their choice, the personal financial costs involved were perceived as a “concern” or “serious concern” by 82% (23/28) of the patients who chose an nr-IPG, whereas the corresponding percentage was 61% (117/192) for the patients who preferred to use an r-IPG. A two-sample binomial test showed that these two sample proportions differed significantly from each other (*z* = 2.2, *p* = 0.031) (Figure 2D).

The proportions of patients who indicated “concern” or “serious concern” at the prospect of further surgeries to replace the battery (Figure 2A), the need for recharging the battery (Figure 2B), and the size of the battery (Figure 2C) was substantial (ranging from 50% of all patients concerned about the prospect of battery recharging to 74% concerning about the need for further surgeries), but the proportions did not differ significantly between the patients with either an r-IPG or an nr-IPG (*z* = 1, *p* = 0.310; *z* = 1.6, *p* = 0.114; *z* = 0.6, *p* = 0.553, respectively).

### Factors Affecting Patient's Choice for Imported or Domestic r-IPG

We examined four factors (patient affordability, the international reputation of DBS manufacturer, DBS remote programming product feature, medical advice) that the patients might have considered when choosing between an imported r-IPG or domestic r-IPG. Among the 192 patients who preferred the use of an r-IPG, 136 (71%) patients chose a device from the international manufacturer (Medtronic) and 56 (29%) chose a device from one of the domestic manufacturers (PINS or SceneRay). A one-sample binomial test indicated that the patient's choice (i.e., 71% of patients favored an imported r-IPG) was not made at random (50%) (*z* = 5.8, *p* < 0.001).

**Figure 3:**
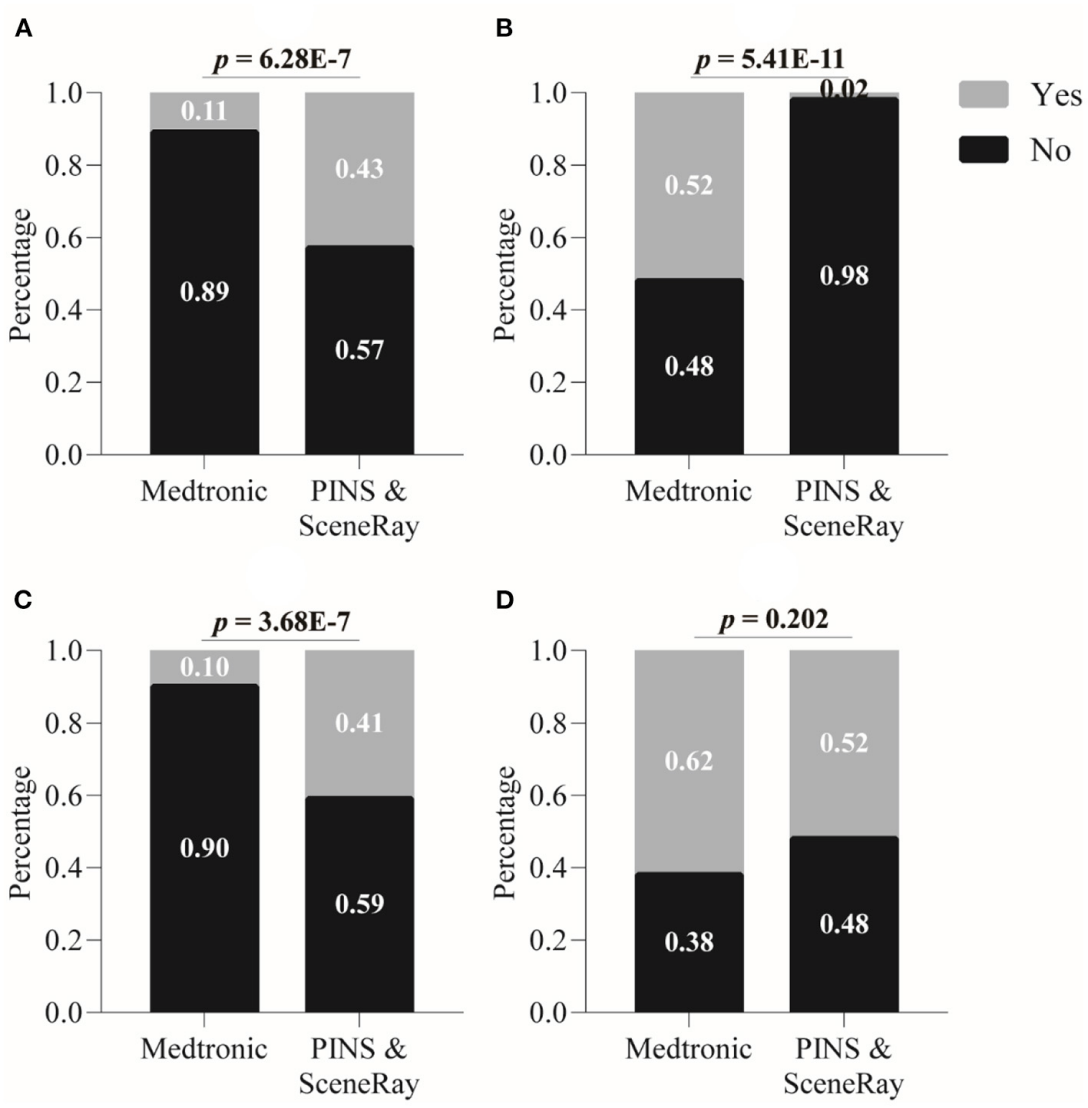
Factors influencing patient's choice between imported and domestic rechargeable implanted pulse generator (r-IPG). **(A)** Budget issue; **(B)** international reputation; **(C)** remote programming feature; and **(D)** convenience of postoperative management. Data are presented as percentages. A *p*-value < 0.05 is considered statistically significant.

Figure 3 illustrates the patients' answers (“yes” or “no”) about whether they considered the above-mentioned four factors in making their decision to use an imported or domestic r-IPG. A two-sample binomial test showed that the proportion of affirmative (“yes”) answers to the question of whether the patients considered their budget was significantly lower among patients who preferred an imported r-IPG (11% of the 136 patients) than those who chose a domestic r-IPG (43% of the 56 patients) (*z* = 5.0, *p* < 0.0001) (Figure 3A). The proportion of affirmative answers regarding the attention to remote DBS programming product features was significantly lower among the patients who preferred an imported r-IPG than those who opted for a domestic one (10 vs. 41%, *z* = 5.0, *p* < 0.0001) (Figure 3C).

In contrast, the proportion of affirmative answers concerning the international reputation of the DBS manufacturer was significantly higher among the patients who chose an imported r-IPG than those who preferred a domestic r-IPG (52 vs. 2%, *z* = 6.5, *p* < 0.0001) (Figure 3B). Finally, the proportion of affirmative answers to the question as to whether medical advice had played a role in the patients' choice was relatively higher in patients who chose an imported brand but did not differ significantly from those choosing a domestic r-IPG (62 vs. 52%, *z* = 1.3, *p* = 0.2002) (Figure 3D).

### Patient Satisfaction

Overall, the majority of patients (88%, 193/220) reported being satisfied with the DBS treatment and the type of IPG device chosen. A minor portion of patients (10%, 22/220) was not satisfied and reported that their expectations of the stimulating effects of DBS treatment were not met. The patient satisfaction rate was 88% among patients with an r-IPG and 86% among patients with an nr-IPG (Table 2). Statistical analysis on the frequency data demonstrated that there was no significant association between patient satisfaction (satisfied vs. unsatisfied) and the type of IPG chosen (r-IPG vs. nr-IPG) (Fisher exact test value = 0.76, *p* > 0.05). Similarly, within the r-IPG patient group, no significant association was observed between patient satisfaction and the manufacturer (imported vs. domestic) of the IPG device (Fisher exact test value = 0.72, *p* > 0.05). Finally, 93% (178/192) of the patients with r-IPGs and 93% (26/28) of the patients with nr-IPGs indicated that they would choose the same type of device again (Table 2). A group of PD patients at late-stage (13 out of 14 = 92.8%) with a median duration of postoperative DBS treatment being 22 (IQR: 3–36) months were satisfied with their choice of r-IPG.

**Table 2:** Patient satisfaction as a function of the type of IPG (*N* = 220).

**Questions**	**Group**	
	**r-IPG**	**nr-IPG**
	**(*n* = 192)**	**(*n* = 28)**
**1. Are you still satisfied with the choice of your device?**		
Yes	169 (88.0%)	24 (85.7%)
No	23 (12.0%)	4 (14.3%)
**1.1. If not, please specify**.		
The stimulating effects did not meet your expectation.	18 (9.4%)	4 (14.3%)
Others	5 (2.6%)	0
**2. Would you choose the same type of your device today?**		
Yes	178 (92.7%)	26 (92.9%)
No	14 (7.3%)	2 (7.1%)

### Patient Experience With Battery Recharging Procedure

The patients' experiences with the battery recharging procedure are summarized in Table 3. The majority of the r-IPG patients (71%, 137/192) were able to perform the battery recharge procedure by themselves. The remaining patients (29%, 55/192), however, were unable to conduct the procedure independently and needed assistance from another person, usually, their spouse, to check and recharge the battery. Furthermore, most patients or their partners (93%) felt confident managing the r-IPG device (Table 3). Almost half of the patients (41%) felt confident managing the r-IPG device within 1 week after being discharged from the hospital. Yet, it took more than 4 weeks for another large portion of the patients (34%) to become proficient in using the device. More than half of the patients (64%) checked the battery every week, and most patients (92%) preferred to recharge when the battery level was still over 50%. The mean interval between subsequent battery recharging procedures was 4.3 days. For most patients (58%), it took about 1 h to complete the recharging procedure. Notably, about 8% of the patients reported that they were unable to recharge the battery on at least one occasion, and more than half of them failed to resolve the technical problem involved. Furthermore, 19% of the patients reported that they forgot to recharge the battery at least once (Table 3).

**Table 3:** Patients' experiences with battery recharging procedure (*N* = 192).

**Questions**	**Number (%)**
**1. Do you feel confident using your r-IPG?**	
No	14 (7.3%)
Yes	178 (92.7%)
**1.1. If yes, how long did it take to feel confident?**	
Within 1 week	78 (40.7%)
1–2 weeks	27 (14.1%)
2–4 weeks	8 (4.2%)
More than 4 weeks	65 (34.0%)
**2. How frequently do you check the battery capacity of your r-IPG?**	
Everyday	35 (18.2%)
Every week	123 (64.1%)
Every 2 weeks	6 (3.1%)
Every 4 weeks	17 (8.9%)
Every year	11 (5.7%)
**3. Have you ever forgotten to recharge your r-IPG?**	
No	156 (81.3%)
Yes	36 (18.7%)
**4. How frequently do you recharge your r-IPG?**	
Everyday	49 (25.5%)
2–4 days	28 (14.6%)
5–7 days	115 (59.9%)
**5. How frequently do you recharge your recharger?**	
Everyday	15 (7.8%)
Every week	104 (54.2%)
Every 2 weeks	31 (16.1%)
Every 4 weeks	37 (19.3%)
Not fixed	5 (2.6%)
**6. At what level of battery capacity do you usually recharge your r-IPG?**	
75–100%	101 (52.6%)
75–50%	76 (39.6%)
<50%	15 (7.8%)
Warning sign	0
**7. How long does recharging usually take?**	
<15 min	9 (4.7%)
15–30 min	37 (19.3%)
30–45 min	35 (18.2%)
45–60 min	40 (20.8%)
More than 60 min	71 (37.0%)
**8. Do you check and recharge your r-IPG yourself?**	
No	55 (28.6%)
Yes	137 (71.4%)
**9. Have you ever been unable to recharge your battery?**	
No	176 (91.7%)
Yes	16 (8.3%)
**9.1. if yes, could you solve the problem on your own?**	
No	11 (68.9%)
Yes	5 (31.2%)

### Mobility in Patients Living With an r-IPG

Most of the patients with an r-IPG (73%) had not traveled since receiving it, and 89% did not return to work postoperatively (Table 4). Among those patients who did travel and went on vacation at least once after surgery (27%), the majority (78%) were able to recharge during the vacation. In daily life, most patients (93%) preferred to sit or lie down while recharging the battery (Table 4).

**Table 4:** Mobility in patients living with a rechargeable IPG (*N* = 192).

**Questions**	**Number (%)**
**1. Have you ever traveled since your DBS surgery?**	
No	141 (73.4%)
Yes	51 (26.6%)
**1.1. If yes, have you ever recharged during the vacation?**	
No	11 (21.6%)
Yes	40 (78.4%)
**2. Do you continue to work after DBS surgery?**	
No	170 (88.5%)
Yes	22 (11.5%)
**2.1. If yes, have you ever recharged during work?**	
No	21 (95%)
Yes	1 (5%)
**3. Are you ambulatory during recharging?**	
No	178 (92.7%)
Yes	14 (7.3%)

## Discussion

To the best of our knowledge, this study included the largest cohort of patients with PD (*N* = 220) to report satisfaction with DBS treatment and the choice of IPG. In this study, most patients (87%) preferred the use of r-IPGs over nr-IPGs for their DBS treatment. Most patients (65%) also preferred an IPG device from an imported brand (Medtronic) over a domestic IPG (PINS or SceneRay). The patients' choice (i.e., 87% chose r-IPGs and 65% chose the imported brand) was significantly different from what would be expected by chance (50%), indicating that the patients had made active, deliberate binary choices among the different options given to them.

The patients accessed information about the DBS treatment for PD prior to their referral to specialized DBS medical care in three primary ways (electronic media, medical advice, word-of-mouth from other patients). The patients' budget for DBS treatment turned out to be the major factor that affected their choice to use either an r-IPG or nr-IPG device. Patients with a relatively low budget tended to choose a cheaper, nr-IPG device. Patient's affordability, international reputation of the manufacturer, and the remote programming product feature also affected the choice of the manufacturer of the IPG device (imported or domestic). The patients' choices were not affected by the medical advice given by their primary care physicians. These results highlighted the role of the patient's economic status as a major non-clinical factor in choosing how the treatment was conducted (10).

The majority of patients (88%) reported being satisfied with the DBS treatment effects and the type of IPG device used. This result aligned with a 3 month follow-up study in patients with movement disorders (*N* = 21) treated with DBS using r-IPGs (9). This follow-up study reported an overall patient satisfaction rate of 83%. In a retrospective study, similar favorable patient experiences were observed in a group of patients with movement disorders (*N* = 35) whose DBS surgeries (using r-IPGs) had been completed for 21 months on average (5). In keeping with the latter study, most patients with an r-IPG in the present study were also able to learn the recharging procedure easily and rapidly. Additionally, no negative correlation was found between age and a patient's ability to handle the recharging procedure (5), although ~30% of patients with an r-IPG did require help from caregivers in our study. Interestingly, several patients only checked their batteries every year, meaning that they recharged the IPG frequently but did not check the IPG status as frequently. In most cases, power depletion did not cause irreversible consequences. Although it was not reported in these participants, there was one patient who got aspiration pneumonia during the power-off period.

Inability to recharge the IPG could be annoying and might induce serious adverse consequences. In our study, most problems were caused by malfunctioning power banks that needed to be replaced. One patient reported that the device was shut down unexpectedly, and the program controller was used to restart the IPG. Another case involved an elderly patient, whose new caregiver at the nursing home failed to recharge the IPG because of unfamiliarity.

Research about patient satisfaction by Timmermann et al. described a small prospective patient satisfaction cohort (*N* = 21) during a 3 month follow-up (9). Jakobs et al. reported another small retrospective group (*N* = 35) with a more extensive follow-up (mean value of 21.2 months) (5). Their work together indicates that there was no significant association between the satisfaction rate and the number of training sessions. These two studies included a small number of patients with PD, dystonia, and essential tremor together into the analysis. Similarly, Jakobs et al. also pointed out that the majority of patients who claimed a lack of confidence after the training program had only participated in only one single session. They, therefore, proposed at least two training sessions for patients (5). In our study, 178 (92.7%) patients felt confident of using their IPG, and such a high rate of satisfaction was probably because our patients and their caregivers were encouraged to participate in two training sessions: one before discharge and one at 1 week after discharge. Patient satisfaction was reported by 31 patients in which 12 received r-IPGs as their initial implants (11). Interestingly, these patients were more satisfied with their IPGs compared with patients who previously had an nr-IPG implanted. In our study, 26 patients had IPG replacement surgery, and 18 out of 26 patients switched from an nr-IPG to an r-IPG and were satisfied with the results. The remote programming function, decreased chances of IPG replacement were the driving force for these 18 patients to change IPG type. The reasons for the eight patients who did not undergo the switch of IPG type were primarily economic concerns and inabilities in DBS self-management. This study did not explore the satisfaction rates among different products but the therapy efficacy was found to be the dominating factor contributing to patients' general satisfaction.

Our results demonstrate that patient affordability had a significant impact on the choice of IPG type in Chinese PD patients. It was estimated that more than 223,000 EUR could be saved over nearly 8 years in 1,499 patients if they were all implanted with r-IPG instead of nr-IPG (12). However, reimbursement policies differ across countries and cities. DBS therapy remains a substantial financial burden for many families as DBS electrodes and IPG are only partly reimbursed by insurance in China. The impact of insurance policies as well as other factors such as the individual life expectancy should be elucidated in future cost-effective investigations.

Previous studies raised the challenging question of how device-aided treatment could be managed in late-stage PD patients (13, 14). In our study, 92.8% of late-stage PD patients were satisfied with the choice of r-IPG. In the meantime, only one patient complained about the tediousness of recharging the battery. The IPG replacement may require an in-hospital admission of a few days and a surgical intervention under total/local anesthesia depending on the patient's compliance. Using the r-IPG could avoid IPG-replacement for these late-stage PD patients, and this was the main reason why r-IPD was preferred by the PD caregivers in our study. Most late-stage PD patients in the study lived at home with their family members, such as a spouse, children, and the r-IPG management was taken care of by their family.

We recognize some limitations in this study. First, as a cross-sectional study, we were not able to identify how the patient's satisfaction developed over time as the disease progressed. Second, the high costs of the DBS treatment could be a financial burden for many families in China which might result in a selection bias. In addition, socioeconomic factors are largely different across regions. Thus, the result of our study may only be generalizable in well-developed regions in China. The education level of the PD patients could also be explored in the future. Finally, in this study, we only analyzed the patients' attitude toward different types of IPG and the recharging process but not an extended assessment for other aspects such as the LEDD. Given the necessity of clinical improvement and the importance of patient satisfaction, we believe that further long-term follow-up studies are needed.

## Conclusion

In conclusion, we found that most patients were satisfied with their choice of IPG. The financial status of patients and the remote programming product feature were the two most critical factors in their decision. The skill required to use a rechargeable IPG was also easily mastered by most patients.

## Data Availability Statement

The original contributions presented in the study are included in the article/Supplementary Material, further inquiries can be directed to the corresponding authors.

## Ethics Statement

The studies involving human participants were reviewed and approved by Ruijin Ethical Committee. The patients/participants provided their written informed consent to participate in this study.

## Author Contributions

KA, CZ, and DL: study conception and design. XQ, TP, ZL, and YW: data collection. ZL and YW: data analysis. XQ, ZL, and CZ: draft writing. All authors critical comments.

## Conflict of Interest

DL and CZ have received honoraria and travel expenses from Medtronic, PINS, SceneRay. BS received research support from PINS and SceneRay (donated devices). The remaining authors declare that the research was conducted in the absence of any commercial or financial relationships that could be construed as a potential conflict of interest.
