# Corrigendum: Fixed-life or rechargeable battery for deep brain stimulation: preference and satisfaction in Chinese patients with Parkinson's disease

**DOI:** 10.3389/fneur.2023.1309820

**Published:** 2023-11-07

**Authors:** Xian Qiu, Tingting Peng, Zhengyu Lin, Kaiwen Zhu, Yuhan Wang, Bomin Sun, Keyoumars Ashkan, Chencheng Zhang, Dianyou Li

**Affiliations:** ^1^Department of Nursing, Ruijin Hospital, Shanghai Jiao Tong University School of Medicine, Shanghai, China; ^2^Department of Neurosurgery, Ruijin Hospital, Shanghai Jiao Tong University School of Medicine, Shanghai, China; ^3^Department of Neurosurgery, King's College Hospital, London, United Kingdom

**Keywords:** deep brain stimulation, movement disorders, patient satisfaction, Parkinson's disease, rechargeable implantable pulse generator

In the published article Jakobs M, Kloß M, Unterberg A, Kiening K. Rechargeable internal pulse generators as initial neurostimulators for deep brain stimulation in patients with movement disorders. *Neuromodulation*. (2018) 21:604–10. doi: 10.1111/ner.12748 was not cited in the article. The citation has now been inserted in **Materials and Methods**, Survey questionnaire, paragraph 1 and should read:

“An internet-based survey questionnaire (powered by www.wjx.cn) was developed to evaluate the patients' perceptions, expectations, and experiences with the DBS treatment and the IPG device they chose (r-IPGs vs. nr-IPGs and international vs. domestic brands). The questionnaire was designed with reference to the work of Jakobs et al. (5), and were adjusted according to the situation of Chinese patients. The questionnaire focused on the patients' satisfaction with the DBS treatment and the IPG of choice as well as four aspects of possible concern (patient affordability, prospect of further required surgeries to replace the battery, requirement for recharging the battery, and battery size) and the sources of information (e.g., clinical advice, electronic media) the patients considered in making decisions. Several extra questions were included in the questionnaire for patients with r-IPGs to collect their experiences with the battery recharging procedure, including how often they checked the battery capacity and the frequency, duration, and ease of recharging, as well as the occurrences that they forgot or were unable to recharge. The patients indicated their answers using a forced-choice dichotomous format or a 4- or 5-point Likert scale. The questionnaire was distributed via the online chat platform WeChat. Participants completed the questionnaire after having received at least 8 months of DBS treatment. In most cases, it took no more than 30 min to complete the questionnaire.”

The authors apologize for this error and state that this does not change the scientific conclusions of the article in any way. The original article has been updated.

