# Supplementary figures and images for: Fixed-Life or Rechargeable Battery for Deep Brain Stimulation: Preference and Satisfaction in Chinese Patients With Parkinson's Disease
Source: Front Neurol. 2021 Jun 15;12:668322. doi: 10.3389/fneur.2021.668322 (PMC8239223; doi:10.3389/fneur.2021.668322)

**Supplementary Figure 1.**

**
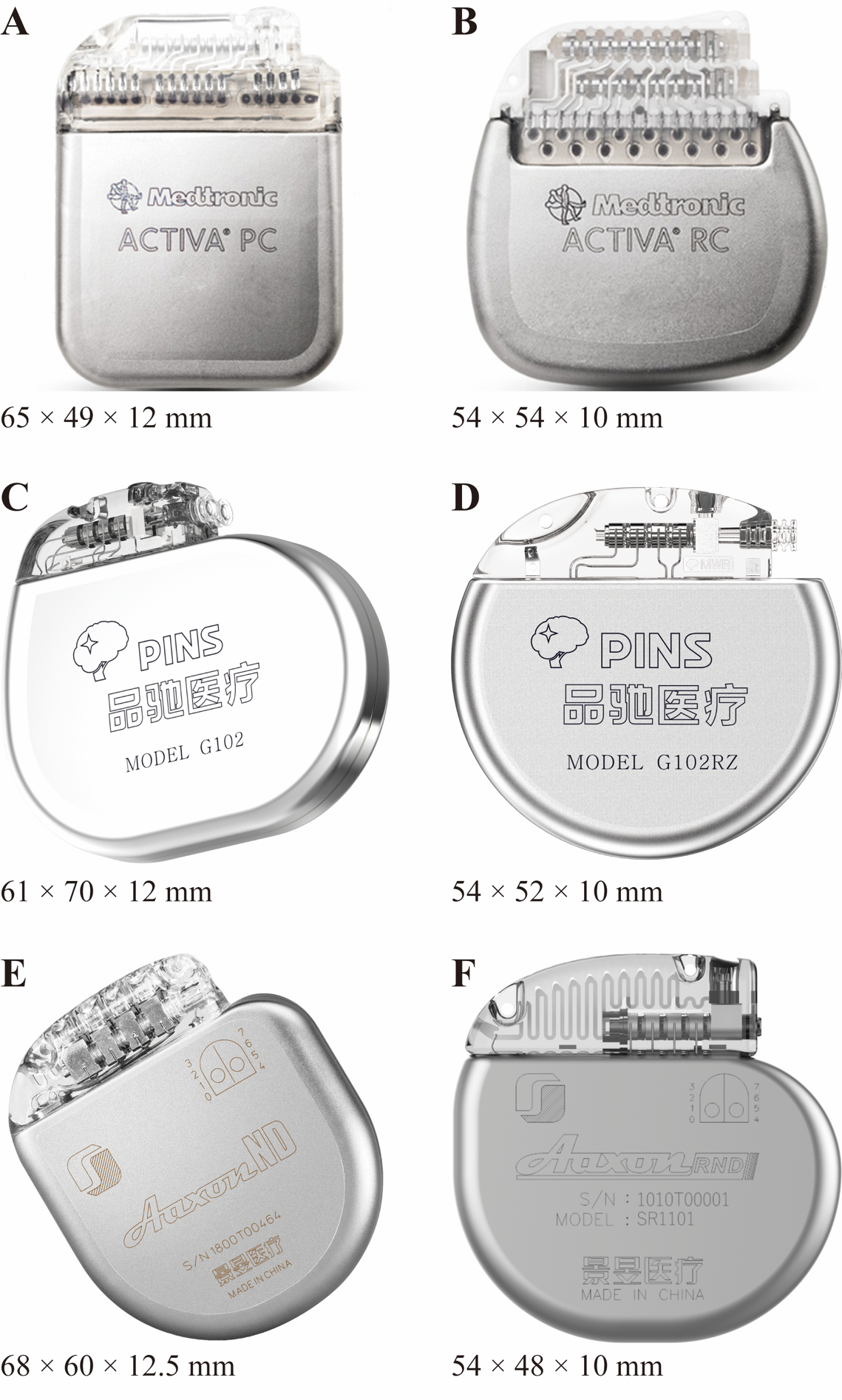
**

Supplement: Supplementary file 1 [file Data_Sheet_1.docx]
